# The Effects of Transdermal Estrogen Delivery on Bone Mineral Density in Postmenopausal Women: A Meta-analysis

**Published:** 2017

**Authors:** Fatemeh Abdi, Hamid Mobedi, Farhad Bayat, Nariman Mosaffa, Mahrokh Dolatian, Fahimeh Ramezani Tehrani

**Affiliations:** a *Ph.D Candidate, Student Research Committee, Nursing and Midwifery Faculty, Shahid Beheshti University of Medical Sciences, Tehran, Iran.*; b *Pharm.D.,Ph.D, Biomaterials Department, Iran Polymer and Petrochemical Institute, Tehran, Iran.*; c *Pasteur Institute of Iran,Research and Production Complex, Tehran, Iran.*; d *Professor, Department of Immunology, Faculty of medicine, ShahidBeheshti University of Medical Sciences, Tehran, Iran.*; e *Assistant Professor, Department of Reproductive Health, Nursing and Midwifery Faculty, ShahidBeheshti University of Medical Sciences, Tehran, Iran.*; f *Professor, Reproductive Endocrinology Research Center, Research Institute for Endocrine Sciences, ShahidBeheshti University of Medical Sciences, Tehran, Iran.*

**Keywords:** Menopause, Bone mineral density, Transdermal estrogen

## Abstract

Due to its minimal systematic adverse effects, transdermal estrogen is widely used for the prevention of osteoporosis in postmenopausal women. The present meta-analysis aimed to clarify the effects of transdermal estrogen on bone mineral density (BMD) of postmenopausal women. Studies were identified by searching electronic databases including Cochrane Library, MEDLINE, Embase , and CINAHL databases, and also the Sciences Citation Index. Systematic review of articles was published between January 1989 to February 2016.Reference lists of the included articles were also evaluated and consultations were made with relevant experts. While 132 studies included the desired keywords, only nine clinical trials met the inclusion criteria and were finally reviewed. The pooled percent change in BMD was statistically significant in favor of transdermal estrogen. According to resulting pooled estimate, lumbar spine BMD one and two years after transdermal estrogen therapy was respectively 3.4% (95% CI: 1.7-5.1) and 3.7% (95% CI: 1.7-5.7) higher than the baseline values. The test for heterogeneity was not statistically significant based on the I^2^ heterogeneity index. One-two years of transdermal estrogen delivery can effectively increase BMD and protect the bone structure in postmenopausal women.

## Introduction

Menopause predisposes women to osteoporosis is a major public health issue (1). Due to concerns about women’s bone health, several efforts have been made to effectively prevent or treat osteoporosis in postmenopausal women all around the world(2-4). Reduced estrogen levels during menopause can increase the risk of trabecular bone loss and lead to multiple bone fractures (5). According to the results of the National Osteoporosis Risk Assessment (NORA) study (6), 7.2% of the 200160 assessed postmenopausal women suffered from undiagnosed osteoporosis that predisposed them to a four-fold increased risk of bone fractures. Bone fractures due to estrogen deficiency cause disability in about two-thirds of women and increase the risk of mortality during the year after diagnosis by 20% (7). Therefore, not only bone loss prevention, but also timely diagnosis of osteoporosis is required to decrease the risk of disability and life-threatening events in postmenopausal women. Hormone replacement therapy (HRT) has thus been recommended as a proper approach to prevent bone loss during the postmenopausal period (8, 9). Steroidal hormone use has been found to change bone mineral density (BMD) in women (10). Despite its advantages, HRT might increase the risk of gall-bladder stones, endometrial cancer (especially in women with an intact uterus), and breast cancer (following estrogen therapy). Such disadvantages and limitations have raised serious concerns which have not been fully resolved. 

The metabolic and therapeutic effects of estrogens depend on their type, dosage, form, and route of administration (11). Several alternative therapeutic options have been applied to provide optimal level of estrogen and reduce the risk of bone loss in the postmenopausal period (12, 13). Some systematic reviews and meta-analyses have confirmed the beneficial effects of bisphosphonates on preserving BMD and decreasing the risk of early osteoporosis in postmenopausal women (14, 15). However, gastrointestinal complications of these agents limit their use (16). Furthermore, while some selective estrogen receptor modulators, such as raloxifene, have been applied to increase bone turnover by elevating BMD, their use has been hindered by the elevated risk of thromboembolic events following their application (13). 

Delivery of estrogens, either alone or in combination with progesterone, by different routes is currently the most accepted method for the prevention of osteoporosis in postmenopausal women (17). Although oral estrogens have long been the commonest regimen for the mentioned purpose (18, 19), transdermal estrogen delivery has recently received growing attention since it can provide its therapeutic effects by a very low dose of hormones and with the fewest side effects. The possibility of gradual delivery of hormones (20-22), particularly through novel nanoparticle delivery systems (23), along with beneficial effects on coagulation processes and lipid metabolism (11) are other advantages of this method. Ultra low-dose hormone therapy can alleviate the clinical symptoms of menopause by ensuring effective protection against the postmenopausal reduction in BMD (24).However, limited knowledge is presently available on the advantages and disadvantages of transdermal estrogen therapy in postmenopausal women (22). To the best of our knowledge, no systematic review has evaluated the effects of transdermal estrogen delivery on BMD in menopausal women. Prior studies have used transdermal estrogen therapy on BMD, but results have been inconsistent partly because of limited statistical power. The objective of the present meta-analysis was to evaluate the efficacy of transdermal estrogen therapy in maintaining BMD among postmenopausal women. 

## Methods

*Literature Search strategy*

The methods of the systematic review were specified in advance and documented in a published protocol in the Prospective Register of Systematic Reviews (PROSPERO).To ensure the rigor of this meta-analysis, we designed and reported it adhering to the criteria set out by PRISMA statement. Relevant clinical trial studies were identified by searching a number of key terms, i.e. “menopause”,“bone mineral density”and “transdermal estrogen” in electronic databases including the Cochrane Library, MEDLINE, Embase, and CINAHL databases, and the Sciences Citation Index. Reference lists of the included articles were also scanned and consultations were made with experts. Clinical trials published in English during January 1989 to February 2016 were reviewed. Articles from any country with relevant information on prevalence were eligible for full review. Studies were excluded if (1) Full text was not available (2) they had case series or case studies(3) studies which did not measure changes in BMD after one-two years and (4)studies without quantitative outcome data.

*Study Selection and Data Extraction*

Studies were included regardless of study quality. First, two researchers (F.A.and F.R.T) independently evaluated all potentially suitable articles based on their title and abstract. Clearly ineligible articles were discarded and full texts of eligible articles were obtained and assessed independently by the two reviewers. Cases of disagreement about the eligibility of studies were resolved by consulting a third reviewer. The papers’ author list, study design, publication date, country, number of participants in each group, drug regimens for hormone therapy, lengh of follow up, and percent change in BMD were extracted. Control groups in trials were receiving either placebo or routine checkup. 

*Quality Appraisal*

The meta-analysis was reported following the PRISMA checklist. All eligible studies were carefully reviewed. Of the 132 studies which included the desired keywords, nine clinical trials met the study criteria and were finally reviewed (25-34).The quality of trials was assessed using the Cochrane Collaboration’s tool for assessing risk of bias in randomized trials. 

*Data analysis*

Pooled changes in BMD by prescribing transdermal estrogen was assessed using the random effects model. This statistical technique weights individual studies by sample size and variance (both within- and between study variance) and yields a pooled point estimate and a 95% confidence interval (CI). This technique was considered as an appropriate pooling technique due to the relative heterogeneity of the source population in each study. The existence of heterogeneity across trials was evaluated using the I^2^ statistic, i.e. P > 0.05 and/or I^2^< 30% indicated homogeneity. Funnel plot and Egger’s test were used to estimate potential publication bias. P-values less than 0.05 from Egger’s test suggested the presence of publication bias. All statistical analyses were performed with STATA 13.1 (StataCorp, College Station, TX).

*Main results*

**Figure 1:**
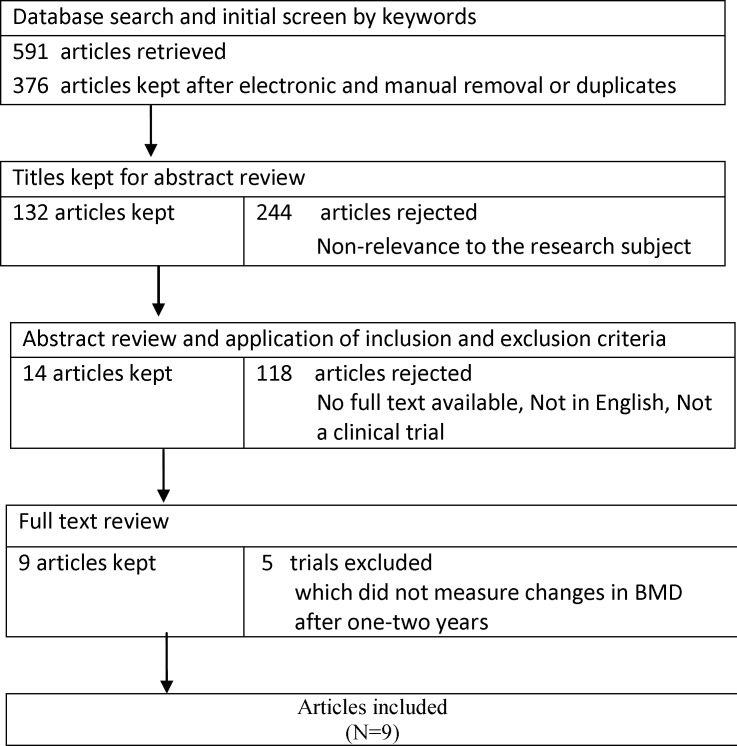
The flow diagram of study selection for the meta-analysis

A flow chart of the literature search and its results is presented in Figure 1. Initially, the search yielded titles and abstracts from all databases. Studies were reviewed in full and nine papers were ultimately included. All these nine studies enjoyed high quality and fulfilled the inclusion criteria, i.e. they assessed the beneficial effects of transdermal estrogen on preserving BMD in postmenopausal women. Since the participants of different studies were followed up for one or two years, we conducted subgroups analysis according to the study follows. The papers were categorized based on their follow-up period. Table 1 summarizes the characteristics of the selected studies. Results of the analysis of quality assessment are shown in Table 2. None of studies had selection bias and attrition bias. There was unclear risk in performance bias in three included studies (25,28,30).Also, two studies had unclear risk in detection bias (31) and reporting bias(28). A total of 643 women were assessed to receive transdermal estrogen. All of studies had control groups receiving either placebo or other routine checkups. Six studies were published after 2001(25-30), and three studies were published before 2000 (31-33).

**Figure 2:**
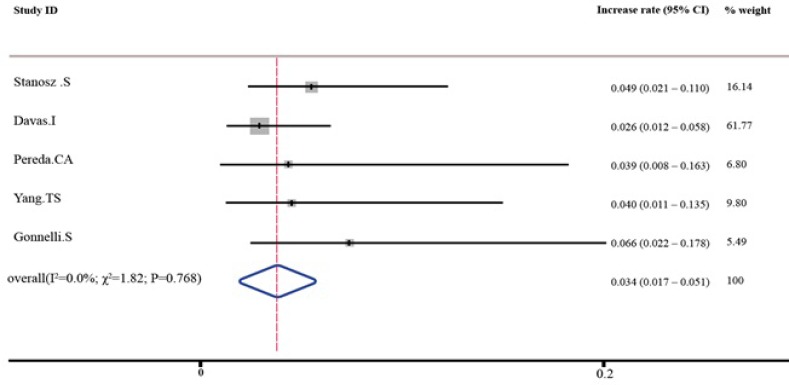
Percent increase in BMD following one year of using transdermal estrogen

**Figure 3:**
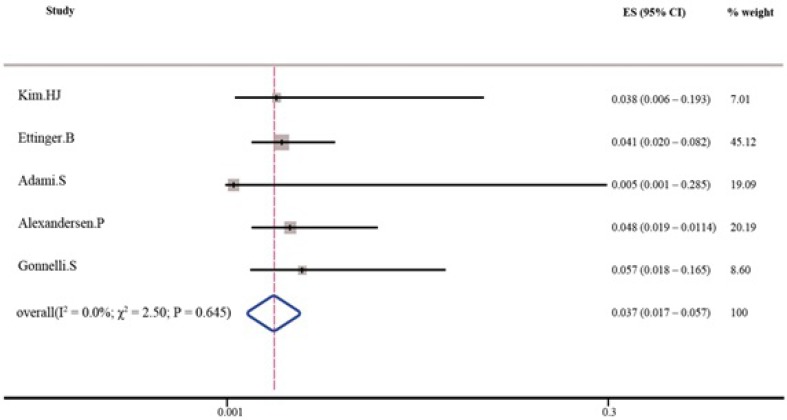
Percent increase in BMD following two years of using transdermal estrogen

**Figure 4:**
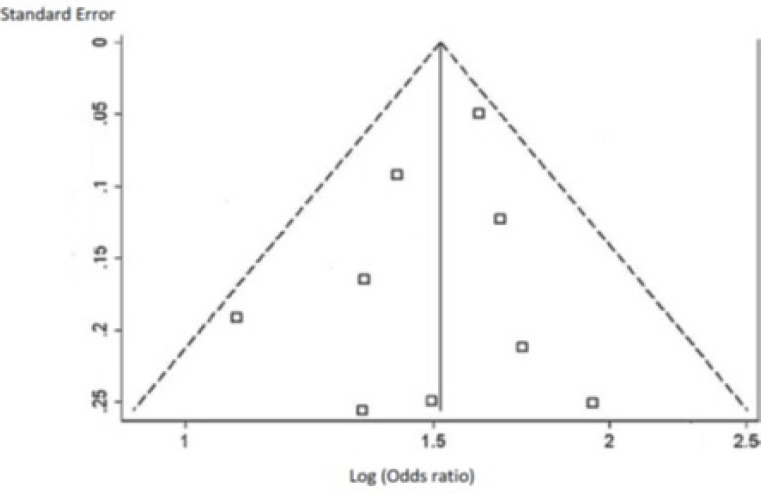
Funnel plot for publication bias**.**

As shown in Tables 3 and 4, the pooled percent change in BMD was statistically significant in favor of transdermal estrogen. According to the resulting pooled estimate, BMD one and two years after transdermal estrogen therapy was respectively 3.4% (95% CI: 1.7-5.1) and 3.7% (95% CI: 1.7-5.7) higher than the baseline values. The test for heterogeneity was not statistically significant in studies with either one-year follow-up (I^2^ = 0.0%; χ^2^ = 1.82; P = 0.768; Figure 2) or two-year follow-up (I^2^ = 0.0%; χ^2^ = 2.50; P = 0.645; Figure 3). Moreover, Egger’s test and developed funnel plots did not suggest publication bias(P = 0.524)(Figure 4).

## Discussion

The present research highlighted an agreement between the reviewed studies with regard to the effectiveness of transdermal estrogen in improving BMD. In fact, one-two years of receiving transdermal estrogen were associated with 3.4%-3.7% increase in BMD. Moreover, in all trials, the drug was well tolerated with no major adverse events. Our analysis also indicated the homogeneity and lack of publication bias across the studies. Minimal side effects and a potential for the gradual delivery of the drug at selective dosages have turned transdermal estrogen therapy as a favorable method for the prevention of osteoporosis in postmenopausal women. Several trials have attempted to assess the efficacy of this method in the improvement and preservation of BMD. Our review of literature confirmed the beneficial effects of transdermal estrogen therapy on increasing BMD during the postmenopausal period. Transdermal estrogen delivery offers some important benefits over oral administration of the drug. Since transdermal estrogen can systemically circulate before reaching the liver, the first-pass metabolism of estrogen in the liver is prevented. Therefore, the desired effects can be obtained with lower doses of the drug (35). This will, in turn, inhibit the overproduction of triglyceride, which is generally seen following the oral administration of estrogen (36-38). In fact, transdermal estrogen delivery has been found to reduce triglyceride levels by 33.7% (38) and is thus believed to decrease the risk of cardiovascular events in postmenopausal women (39, 40). Based on previous research, transdermal estrogen delivery can decrease the incidence of coronary artery disease by reducing systolic blood pressure and vascular resistance while elevating cardiac stroke volume and cardiac output (41-43). In fact, because of simultaneous effects of transdermal estrogen delivery on both BMD preservation and cardioprotection, this therapeutic regimen is now superseding other treatment modalities. However, as mentioned earlier, some long-term side effects of transdermal estrogen delivery, including the increased risk of endometrial and breast cancers, have limited its application. 

Several clinical trials have assessed the efficacy of estrogen-based hormone therapy in reducing the risk of bone fractures, especially in postmenopausal women. However, the efficacy of such treatments may depend on two main factors, i.e. women’s age and the minimal effective dose of estrogen (44). Early studies in this field indicated estrogen doses lower than 0.625 mg to be practically ineffective in decreasing bone fracture risk (44, 45). Later studies, however, found estrogen doses as low as 0.3 mg to be capable of preserving BMD during both premenopausal and postmenopausal periods (46). A significant increase in total BMD was also observed in postmenopausal women who took 0.25 mg/day of estrogen for three years (47). Nevertheless, the beneficial effects of transdermal estrogen therapy on the skeletal health may depend on women’s endogenous estrogen levels before treatment (48).

**Table 1:** Review of studies related to the effects of transdermal estrogen delivery on BMD in postmenopausal women

Author	Year	Country	Study design	participants	Drug regimens	Follow-up	Increase in BMD
Kim(25)	2014	South Korea	Comparative retrospective clinical trial	N=149 (100: HRT) (49: control)	Transdermal estrogens were a patch (estradiol 1.5 mg patch, Estran-50 patch, twice a week, n = 21) or gel (0.1% estradiol gel, 1.5 mg once daily	2 years	4.9% (lumbar spine), 4.2% (hip)
Stanosz(26)	2009	Poland	Randomized controlled trail	N=75 (25: HRT) (25: control) (25:HST)	Micronized 17β- estradiol (molar mass, 272.39 g/mol) in the form of patches at increasing-decreasing doses (25, 50, 75, and 75 μg per dose)and progesterone in the second phase of the therapeutic cycle.	1 year	3.8% (lumbar spine L2-L4 (g/cm2)
Ettinger(27)	2004	USA	Randomized,placebo-controlled trial	N=417 (208: HRT) (209: control)	Unopposed transdermal estradiol at 0.014 mg/day	2 years	2.6% (lumbar spine) 0.4% (total hip)
Davas(28)	2003	Turkey	Comparative prospective clinical trial	N=160 (80: HRT) (80: routine checkups)	Transdermal estrogen 0.05 mg twice weekly, and daily MPA, 5 mg orally or and alendronate, 10 mg orally	1 year	4.1% (lumbar spine)
Pereda (29)	2002	UK	Randomized placebo-controlled trial	N=21 (10: HRT) (11: routine checkups)	25 mg estradiol implant inserted subcutaneously beneath the skin of the abdomen	1 year	5.4% (lumbar spine) 6.0% (total hip) 3.7% (femoral neck
Yang (30)	2007	Taiwan	Comparative Prospective clinical trial	N=120 (90: HRT) (30: routine checkups)	Transdermal administration of estradiol gel at a daily dosage of 1.25, 2.5 and 5.0 g (containing 0.75, 1.5, and 3 mg of 17beta-estradiol/day)	1 year	4.8% (lumbar)
Adami(31)	1989	Italy	Randomised controlled trial	N=68 (34: HRT) (34:control )	Transdermal estradiol (ESTRADERM TTS-50), 50 micrograms/day and medroxyprogesterone (10 mg/day for 12 days)	2 years	4.3% (lumbar)
Alexandersen(32, 34)	1999	Denmark	Randomized placebo-controlled trail	N=100 (51: HRT) (49: control)	Transdermal 17beta-estradiol, releasing 50 microg/day; plus oral norethisterone acetate (NETA), 1 mg/day	2 years	4.0% (spinal)
Gonnelli(33)	1997	Italy	Randomized controlled trail	N=90 (45: HRT) (45: control)	transdermal estrogen( 0.05 mg/day 17 beta-estradiol) and calcium	1 year 2 years	5.7% (lumbar) 6.6% (lumbar)

HRT: Hormone replacement therapy; MPA: Medroxyprogesterone acetate; HST: Hormonal supplementary therapy; NETA: Norethisterone acetate

**Table 2:** Quality appraisal by Cochrane Collaboration’s tool for assessing risk of bias

No.	Domain	Selection bias	Performance bias	Detection bias	Attrition bias	Reporting bias	Other bias
	Reference						
1	Kim,2014	+	?	+	+	+	?
2	Stanosz,2009	+	+	+	+	+	?
3	Ettinger,2004	+	+	+	+	+	?
4	Davas,2003	+	?	+	+	?	?
5	Pereda,2002	+	+	+	+	+	?
6	Yang,2007	+	?	+	+	+	?
7	Adami ,1989	+	+	?	+	+	?
8	Alexandersen,1999	+	+	+	+	+	?
9	Gonnelli,1997	+	+	+	+	+	?

Key: Low risk: **+; **Unclear risk:? ; High risk: **-**

**Table 3:** Increase in BMD following one year of using transdermal estrogen

**Study **	**Increase rate (95% confidence interval)**	**% weight**
Stanosz(26)	0.049 (0.021 – 0.110)	16.14
Davas(28)	0.026 (0.012 – 0.058)	61.77
Pereda (29)	0.039 (0.008 – 0.163)	6.80
Yang (30)	0.040 (0.011 – 0.135)	9.80
Gonnelli(33)	0.066 (0.022 – 0.178)	5.49
Fixed pooled	0.034 (0.017 – 0.051)	100

**Table 4:** Increase in BMD following two years of using transdermal estrogen

**Study**	**Effect size (95% confidence interval)**	**% weight**
Kim (25)	0.038 (0.006 – 0.193)	7.01
Ettinger(27)	0.041 (0.020 – 0.082)	45.12
Adami(31)	0.005 (0.001 – 0.285)	19.09
Alexandersen(32)	0.048 (0.019 – 0.0114)	20.19
Gonnelli(33)	0.057 (0.018 – 0.165)	8.60
Fixed pooled	0.037 (0.017 – 0.057)	100

Finally, while transdermal estrogen therapy did not change women’s lipoprotein profile (49), it favorably modified platelets hemostasis and reversed the adverse effects of menopause (50). Moreover, transdermal estrogens might be safe with respect to thrombotic risk (51, 52). In conclusion, it seems that one-two years of transdermal estrogen delivery can increase bone density, preserve BMD, and successfully protect the bone structure in postmenopausal women. It can thus prevent single or multiple bone fractures and their consequent disability and poor quality of life in older women. There are limitations of this study that should be considered. There was a lack of articles that met the inclusion criteria for meta-analysis. As with other similar meta-analyses; this study is restricted by the heterogeneity of the included trials. 

*Implications of the systematic review for clinical practice:*

Based on our findings, there is a need to revise recommendations about the effects of transdermal estrogen delivery on BMD in postmenopausal women. Transdermal estrogen can provide adequate skeletal loading and successfully protect the bone structure and reduce the risk of fractures in older women.
